# Causes of death in Tonga: quality of certification and implications for statistics

**DOI:** 10.1186/1478-7954-10-4

**Published:** 2012-03-05

**Authors:** Karen Carter, Sione Hufanga, Chalapati Rao, Sione Akauola, Alan D Lopez, Rasika Rampatige, Richard Taylor

**Affiliations:** 1 School of Population Health, University of Queensland, Herston (Brisbane), Queensland, Australia; 2 Ministry of Health, Tonga, Australia; 3 Western Health, Melbourne, Australia; 4 School of Public Health and Community Medicine, University of New South Wales, Randwick (Sydney), NSW, Australia

**Keywords:** Mortality, Cause of death, Noncommunicable Diseases, Medical record review, Death Certification, Tonga, Pacific Islands

## Abstract

**Background:**

Detailed cause of death data by age group and sex are critical to identify key public health issues and target interventions appropriately. In this study the quality of local routinely collected cause of death data from medical certification is reviewed, and a cause of death profile for Tonga based on amended data is presented.

**Methods:**

Medical certificates of death for all deaths in Tonga for 2001 to 2008 and medical records for all deaths in the main island Tongatapu for 2008 were sought from the national hospital. Cause of death data for 2008 were reviewed for quality through (a) a review of current tabulation procedures and (b) a medical record review. Data from each medical record were extracted and provided to an independent medical doctor to assign cause of death, with underlying cause from the medical record tabulated against underlying cause from the medical certificate. Significant associations in reporting patterns were evaluated and final cause of death for each case in 2008 was assigned based on the best quality information from the medical certificate or medical record. Cause of death data from 2001 to 2007 were revised based on findings from the evaluation of certification of the 2008 data and added to the dataset. Proportional mortality was calculated and applied to age- and sex-specific mortality for all causes from 2001 to 2008. Cause of death was tabulated by age group and sex, and age-standardized (all ages) mortality rates for each sex by cause were calculated.

**Results:**

Reported tabulations of cause of death in Tonga are of immediate cause, with ischemic heart disease and diabetes underrepresented. In the majority of cases the reported (immediate) cause fell within the same broad category as the underlying cause of death from the medical certificate. Underlying cause of death from the medical certificate, attributed to neoplasms, diabetes, and cardiovascular disease were assigned to other underlying causes by the medical record review in 70% to 77% of deaths. Of the 28 (6.5%) deaths attributed to nonspecific or unknown causes on the medical certificate, 17 were able to be attributed elsewhere following review of the medical record. Final cause of death tabulations for 2001 to 2008 demonstrate that noncommunicable diseases are leading adult mortality, and age-standardized rates for cardiovascular diseases, neoplasms, and diabetes increased significantly between 2001 to 2004 and 2005 to 2008. Cause of death data for 2001 to 2008 show increasing cause-specific mortality (deaths per 100,000) from 2001-2004 to 2005-2008 from cardiovascular (194-382 to 423-644 in 2005-2008 for males and 108-227 to 194-321 for females) and other noncommunicable diseases that cannot be accounted for by changes in the age structure of the population. Mortality from diabetes for 2005 to 2008 is estimated at 94 to 222 deaths per 100,000 population for males and 98 to 190 for females (based on the range of plausible all-cause mortality estimates) compared with 2008 estimates from the global burden of disease study of 40 (males) and 53 (females) deaths per 100,000 population.

**Discussion:**

Certification of death was generally found to be the most reliable data on cause of death in Tonga available for Tonga, with 93% of the final assigned causes following review of the 2008 data matching those listed on the medical certificate of death. Cause of death data available in Tonga can be improved by routinely tabulating data by underlying cause and ensuring contributory causes are not recorded in Part I of the certificate during data entry to the database. There is significantly more data on cause of death available in Tonga than are routinely reported or known to international agencies.

## Introduction

Cause of death data by age group and sex is critical information both to identify key public health issues contributing to premature mortality and to design interventions that are appropriately targeted. Several studies have previously noted that there is an important gap in availability of data on mortality and cause of death patterns from the Pacific Islands [1,2], and the World Health Organization (WHO) mortality database currently lists Tonga as having no cause of death data available [3].

Tonga is a Pacific Island country with an estimated population of 103,000 (2009) [4], of which 38% are under 15 years of age [4]. Seventy percent of the population reside on the main island of Tongatapu [4]. Recent (2005 to 2009) estimates of adult mortality (probability of dying between ages 15 and 59 years inclusive) based on reconciled empirical data from the Ministry of Health (MoH) and Civil Registration office, are nearly three times that for Australia and New Zealand [5]. Estimated life expectancy (LE) from these data is between 60.4 to 64.2 years for males and 65.4 to 69.0 years for females [6] when assessed for underenumeration, several years lower than previously reported [5-7]. Given the high level of premature adult mortality and the impact on LE, it is critical that health planners have access to accurate information on causes of death.

Health services in Tonga are provided through a national hospital, three outerisland hospitals, 11 reproductive health centers, and 15 community health centers [8]. Prior to 2007, medical certification of death was conducted by the attending doctor for deaths occurring at health facilities or by the treating doctor for deaths in community if requested by the family. In January 2007, the Tongan MoH introduced a new policy on reporting deaths as part of a comprehensive effort to improve vital registration [9]. This policy requires all deaths to be certified by a medical practitioner. Tonga uses a medical certificate of death (hereafter referred to as the medical certificate) that is consistent with the international standard medical certificate of death from the 10^th ^version of the International Classification of Diseases (ICD-10) [10]. This lists the cause of death sequence starting with the immediate cause of death (the condition that directly resulted in the death) and working backwards in Part I of the certificate and contributory causes (conditions present at the time of death, but which did not directly cause the death) in Part II (Additional file 1).

In accordance with ICD-10, underlying cause of death (the condition that started the chain of events that resulted in the death) is generally selected by working backwards from the immediate cause to the cause on the lowest line in Part I, which is able to directly cause the condition above it. If the cause listed on the lower line is not a direct cause of the condition listed above (for example if cancer was listed below ischemic heart disease), the lower line is not included in the causal sequence and the line above is selected as the underlying cause (ischemic heart disease in the previous example). Modification and selection rules also apply for certain conditions [10]. In Tonga, data from the medical certificates are currently coded line by line (without selecting underlying cause) by medical records staff who have completed a recognized training course in ICD coding and collated in an electronic database. Tabulations are generated from this database as required, and until this study, were based on the immediate cause of death (line 1 of Part I of the certificate). The Country Health Information Profile, developed from local data and published by the Western Pacific Regional Office of WHO, lists only five leading causes of death for Tonga (based on 2007 data) reported for all ages and both sexes combined. These are: 1) Diseases of the circulatory system; 2) Neoplasms; 3) Symptoms, signs, and ill-defined conditions; 4) Diseases of the respiratory system; and 5) Certain infectious and parasitic diseases [8].

This paper examines the accuracy of the locally generated cause of death data for Tonga for 2008 as collected through the medical certificates. Cause of death as recorded on the certificate is compared to information contained in the patients' medical record to assess reported patterns of cause of death and to identify (and correct) any systematic misclassifications that may affect selection of underlying cause of death or analysis of trends. Findings from this assessment were applied to cause of death data from the medical certificates of death for 2001 to 2007, which were then added to the dataset. Proportional mortality by age and sex was calculated and applied to age-specific all-cause mortality for 2001 to 2008 [5] to produce age-, sex- and cause-specific mortality for Tonga and assess trends over time.

## Methods

**Figure 1:**
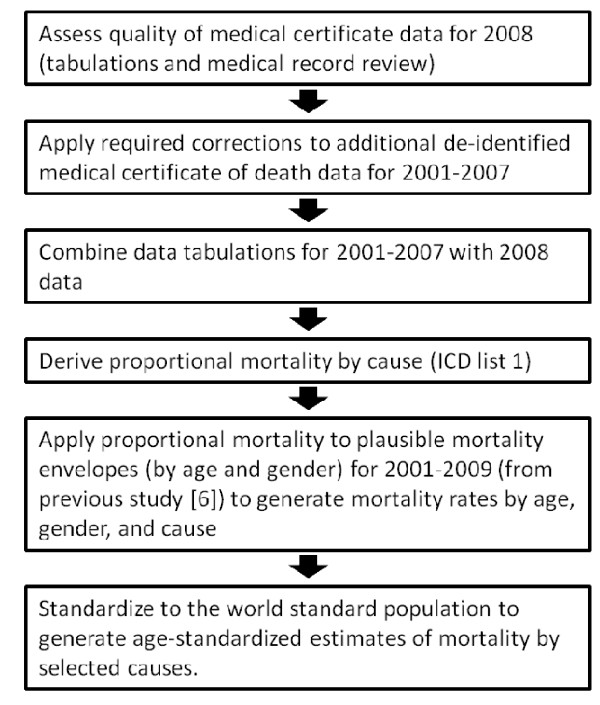
**Overview of study design**.

An overview of the study approach is given in Figure 1.

### Quality review of cause of death data for 2008

Data from the medical certificates were sought from the MoH for all reported deaths in Tonga for 2008 (n = 945). Cause of death data from the certificate (both Part I and Part II) were entered into the database by MoH medical record staff as the ICD-10 code only [10]. During data entry for some deaths, contributory causes listed in Part II of the original medical certificate were entered onto the first available line in the database sequence in Part I. This could erroneously lead to their consideration within the causal sequence recorded in the database, resulting in the possibility that such a contributory cause may be selected as the underlying cause (according to the General Principle for mortality coding in ICD-10), if it could plausibly lead to the condition listed above it.

The quality of population cause of death data obtained through medical certification is directly affected by three stages in the data collection process: original diagnosis and certification of the death, coding of the data from the certificate, and the data entry and tabulation practices used to produce aggregate data. As data from the certificates of death were obtained as an extraction from the MoH database (with cause of death available only as ICD codes), certification and coding could not be examined separately. Deaths for 2008 were therefore examined using: (a) a review of the data entry and tabulation practices; (b) a medical record review; and (c) assignment of a final underlying cause from either the medical certificate of death or medical record based on the best available information.

#### Review of tabulation practices

An underlying cause of death was assigned for each death based on ICD-10 [10]. When the unit record listed more than one cause, the underlying cause of death was assigned assuming no movement of contributory causes into Part I of the certificate. Assigned underlying causes were then reviewed to assess whether the selection of underlying cause would differ if it was assumed that contributory causes had been recorded in empty lines for Part I of the certificate when entered in the database. Coded data for both the immediate and underlying cause of death from the medical certificate of death were aggregated to one of the 103 causes listed in the General Mortality Tabulation List 1 of ICD-10 (hereafter referred ICD list 1) [10]. These were crosstabulated to compare the effect of current MoH tabulation practices (based on immediate cause) on the cause of death profile (Table 1).

**Table 1:** Comparison of original cause of death tabulation based on immediate cause from the medical certificate against underlying cause of death from the medical certificate†: ICD 103 cause list (selected causes), Tonga, 2008

Original tabulation	Underlying cause of death from medical certificate 1																					
	
	1-001- 1-025*	1-012	1-019	1-026	1-052	1-059	1-064- 1-071+	1-065	1-067	1-069	1-072	1-080	1-082	1-084	1-087	1-092	1-093	1-094	1-095	Other	Unknown	Total
1-001 - 1-025*	**3**																					**4**

**1-012**	1	**2**	2		10	3					2		4	2		1				3		**30**

**1-019**			**1**																			**1**

**1-026**			1	**55**																		**56**

**1-052**					**12**																	**12**

**1-064 -** **1-071+**				2	9		**37**	2	5		6			1						1		**63**

**1-067**					6				**28**						1							**35**

**1-069**					2				1	**15**									2			**20**

**1-072**	3			4	4		5			1	**34**											**51**

**1-080**			5	2								**6**										**13**

**1-083**		1											**1**									**2**

**1-084**		1		2										**7**								**10**

**1-092**																**13**						**13**

**1-093**																	**3**					**3**

**1-094**	2						5		1	1			1			1		**10**	1	3		**25**

**1-095**																						**0**

**Other**	1			2	15	1								2						**10**		**31**

**Injury~**																			24			**24**

**Unknown**					1														1		**36**	**38**

**Total**	**10**	**4**	**9**	**67**	**59**	**4**	**47**	**2**	**35**	**17**	**42**	**6**	**6**	**12**	**1**	**15**	**3**	**10**	**29**	**17**	**36**	**431**

†Determined according to ICD principles [10], excluding potential impact of contributory causes (Part II) in the causal sequence (Part I)

Single causes from ICD list 1 are recorded in italics, disease groups are shown in plain text.

1-001-1025 Infectious and parasitic diseases (other than septicemia and viral hepatitis)

*1*-*012 Septicemia*

*1*-*019 Viral hepatitis*

1-026 Neoplasms

*1*-*052 Diabetes*

*1*-*059 Meningitis*

1-064 - 1071 Diseases of the circulatory system (excluding rheumatic, ischemic, and cerebrovascular heart disease)

*1*-*065 Rheumatic heart diseases*

*1*-*067 Ischemic heart disease*

*1*-*069 Cerebrovascular heart disease*

1-072 Diseases of the respiratory system

*1*-*080 Diseases of the liver*

1-084 Diseases of the genitourinary system

1-087 Pregnancy, childbirth, and the puerperium

1-092 Certain conditions originating in the perinatal period

1-093 Congenital malformations, deformations, and chromosomal abnormalities

1-094 Symptoms, signs, and abnormal clinical and laboratory findings not elsewhere classified

1-095 Injury, poisoning, and certain other consequences of external causes

Other All other causes

#### Medical record review

Medical records were sought from the national hospital for all deaths that occurred in 2008 in Tongatapu where proximity to the major hospital suggests that deceased individuals were more likely to have received care at the hospital and therefore have a medical record. Data from the medical record for each death were extracted using a standardized form adapted from similar work in the Philippines [11,12], de-identified, and assigned a study number. Data extraction was completed by qualified nurses who underwent initial training in data extraction procedures based on the written study protocol. Extraction forms were regularly reviewed throughout the project [by KC].

An independent medical doctor was provided with medical record extracts and requested to complete a medical record review form for each death based on the international standard medical certificate of death [13]. Doctors were provided with both written and verbal instruction on completing the medical record review form based on ICD-10 guidelines [10]. Where age at death was less than 60 years and cause of death could not be determined, the record extracts were reviewed by a second independent medical doctor. For each cause of death assigned, the doctor was requested to indicate the level of certainty of the diagnosis (definite, strong certainty, some certainty, and limited certainty) based on the available evidence [12]. Assigned causes were subsequently grouped into two diagnostic evidence categories: high (definite and strong) and low (some and limited) [12].

Underlying cause of death from the medical record review form was then coded according to ICD-10 [10] and matched to the medical certificate of death by study number. Underlying cause of death as noted on both the certificate of death and the medical record review form was crosstabulated using ICD list 1 [10]. Differences between the two underlying causes were classified as either minor (different cause as tabulated using ICD list 1, but same ICD chapter) or major (different ICD chapter), and significant associations in reporting patterns were examined.

#### Assignment of final underlying cause of death

Previous medical record review studies [14-16] have started from the presumption of a reference standard, where one source (the medical record) is assumed to be more accurate than the other (the medical certificate). However, such an assumption was not valid in this study. A final underlying cause of death was assigned for each death based on the best available data, whether from the medical certificate or the medical record. In general, the medical certificate-based underlying cause of death was selected, except in the following instances:

a) where cause on the medical certificate was unknown or coded as nonspecific

b) an injury leading to the death was noted in the medical record, but not mentioned on the death certificate

c) the underlying cause identified from the medical record could have led to the underlying cause on the medical certificate of death

d) for all remaining deaths not due to external causes, the underlying cause from the medical record was considered to be of high certainty

Proportional mortality by cause by age and sex was then tabulated and 95% confidence intervals were calculated using the Poisson distribution.

### Cause of death tabulations, 2001 to 2008

Based on the assessment of deaths in 2008, it was determined that medical records added little to our understanding of underlying cause of death at a population level, particularly for adult deaths. For 2008, 93% of the final assigned causes of death were consistent with the medical certificate of death (see results) following the medical record review. Based on this finding, de-identified data from the medical certificates for all deaths for 2001 to 2007 were added to the 2008 data set. As immediate cause differed from the underlying cause (ICD list 1) in 37% of cases for 2008 (see results), each death (2001 to 2007) was reviewed and assigned an underlying cause of death based on ICD-10 [10], by either assuming no movement of contributory causes into Part I of the certificate or assuming that contributory causes had been recorded in empty lines for Part I, as completed for the 2008 data. This resulted in two separate underlying causes, and tabulations by age group and sex were generated for both variables. Proportional mortality for both tabulations was calculated (with exclusion of ill-defined and unknown deaths) for 2001-2004 and 2005-2008 and applied to estimates of total deaths for Tonga to calculate mortality rates by age, sex, and selected causes (ICD list 1). Total deaths for 2001-2004 and 2005-2009 (the closest available period to 2001-2008) were extracted directly from life tables generated by a recent study that established upper and lower mortality scenarios based on adjusted empirical data reconciled from both MoH and Civil Registry sources [5]. Both underlying cause of death tabulations described above were applied to the upper and lower mortality scenarios from this recent study [5], generating four estimates for total deaths by cause by age group and sex for each period. The upper and lower values from these four estimates were used to generate a plausible range of deaths and mortality rates by cause.

Population by age group and sex was derived from the 1996 [17] and 2006 [7] censuses using exponential interpolation to provide estimates for intermediate and additional years. Age-standardized mortality rates for all ages were then calculated using the world-standard population [18] to examine trends over time (2001-2004 and 2005-2008).

Final cause of death was also classified to the WHO Global Burden of Disease (GBD) Study categories: I (infectious, perinatal, and maternal conditions), II (noncommunicable diseases), and III (external causes) [19]. Proportional mortality by GBD category for males was compared with that derived from Codmod [20] to assess broad plausibility of the results. Codmod is a modeling program that generates expected cause of death distributions [20] from inputs of level of childhood and adult mortality and is based on associations between these parameters as recorded in over 100 years of data from a wide range of countries. This was not performed for female deaths as existing Codmod models [20] do not allow input of the high level of adult mortality estimated in females in Tonga concurrently with the low estimates of childhood mortality [5].

## Results

### Reported deaths

Of 945 deaths recorded in Tonga for 2008 based on reconciled MoH and civil registry data [5], 431 deaths had a certificate recorded in the MoH database; 390 of these were from Tongatapu and therefore had a medical record sought for review. As such, certificates were not available for 54% of the reported deaths (reconciled from the Civil Registry, nursing records, and health information system).

There were 2,310 deaths extracted from the MoH database (based on medical certification) for 2001 to 2004, of which 1,902 cases included cause of death data. For 2005 to 2008, 2,025 cases were extracted from the database, of which 1,885 included cause of death data.

### Quality review of deaths in 2008

#### Review of tabulation practices

Examination of case data for 2008 from the certificates revealed that in 63% of deaths the cause of death originally tabulated on immediate cause was the same as the final underlying cause of death when aggregated to the ICD list 1 [10] (Table 1). However, a substantial proportion of deaths originally tabulated as septicemia (1-012) (93%), cardiovascular diseases (1-064 to 1-071) (25%), respiratory illnesses (1-072) (33%), and liver disease (1-078) (54%) should have been attributed to other causes (Table 1) based on ICD rules [10], reflecting various nonspecific causes, such as heart failure and respiratory arrest, which often appear on the first line of the medical certificate of death. Twelve of the 17 deaths (71%) recorded as "signs, symptoms or ill-defined conditions" should have been attributed to more specific causes (Table 1), even with no further information available from the medical record.

Diabetes (1-052) was notably underrepresented in the original tabulation, with 47 of 59 (80%) deaths for which diabetes was selected as the underlying cause originally assigned to other causes, notably septicemia and cardiovascular disease. Neoplasms (1-026) were also underrepresented in the original tabulations with 12 (18%) cancer deaths assigned to other causes. Although they accounted for only a small number of deaths, some deaths attributed on the medical certificate of death to underlying causes of particular public health importance, such as rheumatic heart diseases and maternal causes, were not apparent in the original tabulation of immediate cause.

Upon review, 36 (8%) deaths for 2008 were found to be potentially affected by the entry of contributory causes from Part II of the certificate into the Part I sequence. In all other deaths application of the ICD rules would result in the same underlying cause (when collated to ICD list 1) [10]. Only one of these deaths involved a death assigned to Infectious Diseases, which would have otherwise been coded to pneumonia (Respiratory Disease); all other deaths occurred amongst those assigned to noncommunicable diseases - both in the original tabulation of immediate cause and selected underlying cause of death. Four of these deaths were minor variations within categories of cardiovascular disease, while 23 cases were diabetes-related deaths that would be assigned to cardiovascular or respiratory diseases if the diabetes code had been recorded in Part II of the certificate (as a contributory cause).

### Medical record review

**Figure 2:**
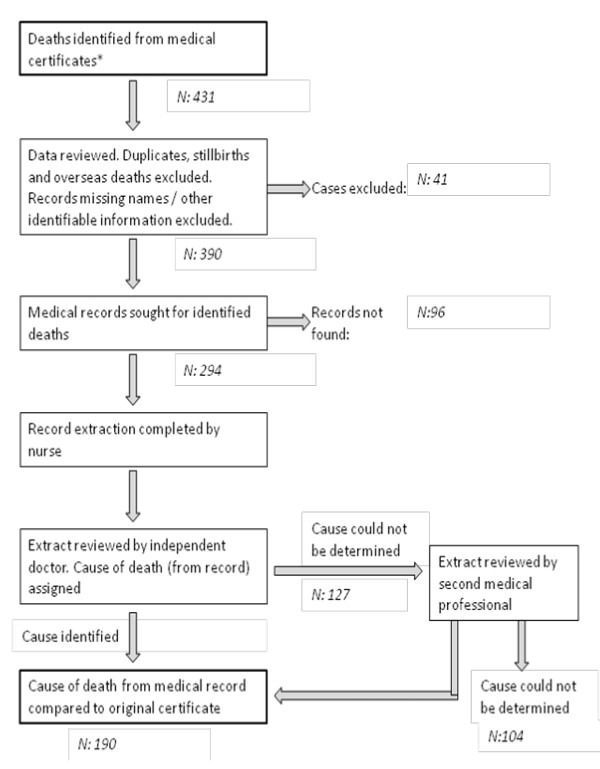
**Flowchart of process for medical record review in Tongatapu**.

Medical records were located for 294 (75%) certified deaths that occurred in Tongatapu (Figure 2) following exclusion of duplicate records and overseas deaths. Cause of death was identified from the medical record for 190 cases, 65% of those with a matching medical record. Diagnosis of underlying cause of death was considered to be of high certainty by the medical reviewer in only 73 (25%) of these records.

The sex and age distribution of deaths for which records could not be located was similar to that for the deaths where a medical record was found, except for older ages ( > 64 years), which had a higher proportion of missing records. Twenty-eight percent of deaths missing a medical record occurred at the national hospital. Total reported deaths followed a similar age and sex distribution as the deaths for which a record was located.

There were 93 major discrepancies (32% of deaths) and 19 minor discrepancies (6% of deaths) identified between the medical death certificate and medical record review, following collation to ICD list 1 and tabulation according to underlying cause of death. If the dataset was limited to deaths with a diagnosis of high certainty, there were 18 major discrepancies (24% of deaths) and four minor discrepancies (5% of deaths). As determined from the medical certificate, 70% of deaths attributed to neoplasms, 77% of deaths attributed to diabetes, and 70% of deaths attributed to cardiovascular disease were assigned to other underlying causes by the medical record review (Table 2). The most common underlying causes of death found by the medical record review to have been originally certified as due to other causes (Table 2) were infectious diseases, in particular septicemia (100% of deaths), cerebrovascular diseases (65% of deaths), and respiratory diseases (71% of deaths). If redistribution of deaths is limited to deaths with a diagnosis of high certainty, nearly all the deaths would be assigned to the same broad ICD category by both the certificate and medical record review.

**Table 2:** Comparison of underlying cause of death from the medical certificate of death† and from the medical record review: ICD 103 cause list (selected causes), Tongatapu, 2008

	Underlying cause of death from medical record review																		
**Underlying cause of death from medical certificate**	1-001- 1-025*	**1-012**	**1-019**	**1-026**	**1-052**	**1-064- 1-071**	**1-067**	**1-069**	**1-072**	**1-080**	**1-082**	**1-083**	**1-084**	**1-092**	**1-093**	**1-095**	**Other**	**Unknown**	**Total**

1-001 - 1-025*	**5**					1					1								**7**

**1-012**	1								1					1					**3**

**1-019**		2	**3**							2								1	**8**

**1-026**	1			**15**		1		2	1	1			1			2	4	23	**50**

**1-052**		1	1		**10**	1	1	8	1	1	3	1	1					14	**43**

**1-059**		1							1			1	1						**4**

**1-064 - 1-071+**						**2**	4		4		1					1	2	18	**32**

**1-065**						1										1			**2**

**1-067**	1	1					**6**	1	1									10	**20**

**1-069**		1		1				**6**									1	3	**12**

**1-072**		2		1		1			**5**									10	**19**

**1-080**																	1	1	**2**

**1-082**											**4**							1	**5**

**1-084**		1											**1**					4	**6**

**1-087**		1																	**1**

**1-092**									1					**11**	1	1			**14**

**1-093**															**3**				**3**

**1-094**									1								1	3	**5**

**1-095**	1				1											**11**		6	**19**

**Other**	2														1	1	**9**	2	**16**

**Unknown**	1			3					1					7	3			**8**	**23**

**Total**	**12**	**10**	**4**	**20**	**11**	**7**	**11**	**17**	**17**	**4**	**9**	**2**	**4**	**19**	**8**	**17**	**18**	**104**	**294**

†Determined according to ICD principles [10], excluding potential impact of contributory causes in the causal sequence

Single causes from the ICD list 1 are recorded in italics, disease groups are shown in plain text.

1-001-1025 Infectious and parasitic diseases (other than septicemia and viral hepatitis)

*1*-*012 Septicemia*

*1*-*019 Viral hepatitis*

1-026 Neoplasms

*1*-*052 Diabetes*

*1*-*059 Meningitis*

1-064 - 1071 Diseases of the circulatory system (excluding rheumatic, ischemic, and cerebrovascular heart disease)

*1*-*065 Rheumatic heart diseases*

*1*-*067 Ischemic heart disease*

*1*-*069 Cerebrovascular heart disease*

1-072 Diseases of the respiratory system

*1*-*080 Diseases of the liver*

1-084 Diseases of the genitourinary system

1-087 Pregnancy, childbirth, and the puerperium

1-092 Certain conditions originating in the perinatal period

1-093 Congenital malformations, deformations, and chromosomal abnormalities

1-094 Symptoms, signs, and abnormal clinical and laboratory findings not elsewhere classified

1-095 Injury, poisoning, and certain other consequences of external causes

Other All other causes

Of the 28 deaths attributed to nonspecific or unknown causes on the medical certificate, 17 (61%) were able to be attributed elsewhere following review of the medical record. Eight (29%) of these unknown cases occurred in children less than 5 years of age and were assigned to perinatal or congenital causes following the medical record review. Evidence for this attribution was definite or strong in each of these cases. Even following medical record review, 11 (6% of all deaths) remained assigned to unknown causes.

#### Assignment of final underlying cause of death

For 208 deaths (71%) for which a medical record was found, the underlying cause of death as determined from the medical record review did not match underlying cause as certified. The medical certificate of death was used in 179 (86%) and the medical record in 29 (14%) of these cases to determine final cause. As noted in the methods, the medical record was used in instances where it added additional information to the causal sequence indicated on the certificate or where an external cause of death was indicated that was not recorded on the medical certificate. The medical records were not considered to be inherently more reliable than the death certificate, as has been assumed in other studies of this type, because of the large proportion of incomplete or missing records, the most recent entry in the record for many cases preceding the death by many years or months, and the poor legibility and completion of records. An overview of the sources of final cause of death data and reasons for selection is shown in Additional file 2; 93% of the final causes assigned matched the underlying cause given by the medical certificate.

Perinatal conditions were the leading cause of death in children (Table 3). Neoplasms were the leading cause of death in adult females, with cardiovascular diseases the leading cause in adult males (Table 4). Diabetes was the second leading cause of death in adult males (19%) and the third leading cause of death in adult females (18%) (Table 4) based on ICD-10, where diabetes is taken as an underlying cause if stated in Part I of the certificate. Half (50%) of all cardiovascular disease was found to be due to ischemic heart disease. Viral hepatitis (4.5% of total adult deaths) was the most frequently reported infectious disease causing mortality in adults.

**Table 3:** Underlying causes of death in Children 0-4 years, Tonga 2008

Cause of death*		Reported deaths	Proportional mortality (%)
1-092	Perinatal conditions	21	36

1-001	Infectious diseases	8	14

1-093	Congenital abnormalities	6	10

1-072	Respiratory diseases	6	10

1-026	Neoplasms	5	9

1-095	External Causes	4	7

1-059	Meningitis	3	5

1-078	Disease of digestive system	2	3

	Other	3	5

* Tabulated according to ICD list 1 [10]. "Infectious diseases" in the table refers to those conditions classified according to chapter 1 of the ICD. Meningitis is coded as a disease of the nervous system under the ICD [10] and is therefore listed separately, while pneumonia and other infectious respiratory illnesses are included in the respiratory diseases chapter

**Table 4:** Underlying causes of death in adults aged 15-64 years, Tonga, 2008

Cause of death*		Males		Females	
		
		Reported deaths	Proportional mortality (%)	Reported deaths	Proportional mortality (%)
**1-064**	**Diseases of the circulatory system**	28	28	16	23

1-067	Ischemic heart diseases	18	18	4	6

1-069	Cerebrovascular diseases	1	1	4	6

1-065	Acute rheumatic fever and chronic rheumatic heart diseases	1	1	1	1

**1-051**	**Endocrine, nutritional, and metabolic diseases **(all diabetes (1-052))	19	19	13	18

**1-095**	**External causes of morbidity and mortality**	18	18	3	4

1-096	Transport accidents	6	6	0	0

1-101	Intentional self-harm	4	4	0	0

**1-026**	**Neoplasms**	15	15	20	28

1-036	Malignant neoplasm of breast	0		3	4

1-037	Malignant neoplasm of cervix uteri	0		2	3

1-039	Malignant neoplasm of ovary	0		2	3

1-031	Malignant neoplasm of liver and intrahepatic bile ducts	3	3	1	1

1-034	Malignant neoplasm of trachea, bronchus, and lung	3	3		0

1-029	Malignant neoplasm of stomach	2	2	2	3

1-030	Malignant neoplasm of colon, rectum, and anus	2	2	2	3

**1-001**	**Certain infectious and parasitic diseases**	10	10	3	4

1-019	Viral hepatitis	6	6	2	3

**1-072**	**Diseases of the respiratory system**	4	4	3	4

**1-078**	**Diseases of the digestive system**	3	3	3	4

**1-084**	**Diseases of the genitourinary system**	2	2	3	4

**1-094**	**Symptoms, signs, and abnormal clinical and laboratory findings, not elsewhere classified**	1	1	1	1

**1-058**	**Diseases of the nervous system**	0	0	3	4

**1-082**	**Diseases of the skin and subcutaneous tissue**	0	0	2	3

**1-087**	**Pregnancy, childbirth, and the puerperium**	0		1	1

	**Other categories**	1	1	0	0

TOTAL		101	100	71	100

* Tabulated according to ICD list 1 [10]. The table shows cause categories by ICD chapter in bold, with important individual cause categories from ICD list 1 shown in small print under their assigned ICD chapter

### Causes of death, 2001 to 2008

**Figure 3:**
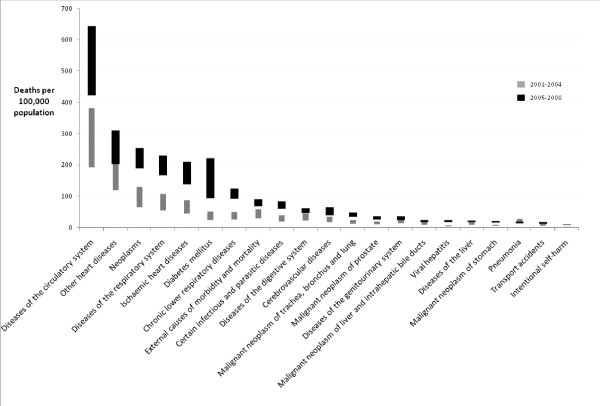
**Age-standardized mortality for males (selected causes) for Tonga, 2001-2009 (plausible range based on upper and lower mortality scenarios)**. Age-standardized mortality rates for all ages were calculated using the world-standard population [18].

**Figure 4:**
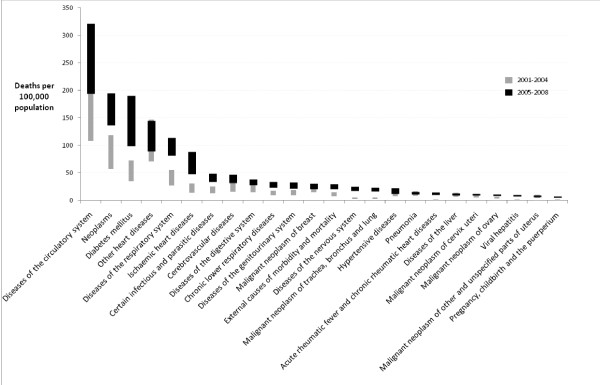
**Age-standardized mortality for females (selected causes) for Tonga, 2001-2009 (plausible range based on upper and lower mortality scenarios)**. Age-standardized mortality rates for all ages were then calculated using the world-standard population [18].

Cause of death data for 2001 to 2008 were compiled from the best available data on underlying cause from either the medical certificate of death or medical record for deaths in 2008, and de-identified medical certificate data for 2001 to 2007. Infectious diseases and neonatal causes were the leading causes of death in 0 to 4 year olds, with injury and external causes the leading cause of death for 15 to 24 year olds. From 25 years of age onwards, proportional mortality from injury and external causes decline with chronic diseases leading proportional mortality for all age groups above 25 years. Age-standardized rates (all ages) for selected diseases are shown in Figures 3 and 4 (Additional file 2), and indicate a significant increase in mortality rates (for males and females) between 2001-2004 and 2005-2008 for a range of noncommunicable diseases, despite the uncertainty in the estimates described in the methods. For 2005 to 2008, the plausible ranges for noncommunicable disease death rates (per 100,000) were cardiovascular diseases (males: 423 - 644 and females: 194 - 321), diabetes (males: 94 - 222 and females: 98 - 190), and lung cancer (males: 34 - 49 and females: 17 - 23). There was a notable increase in age-standardized mortality due to intentional self harm in males, with the age-standardized rate estimated as 9 to 12 deaths per 100,000 in 2005 to 2008.

The overall cause distribution by GBD category from the empirical data for Tongan males for both 2001-2004 and 2005-2008 was very similar to the modeled distribution produced by Codmod [20], which reflects the anticipated cause distribution based on mortality levels as derived from a wide range of previous mortality data sets. However, the Tongan empirical data shows a higher proportion of deaths from noncommunicable diseases in early adult ages than the modeled data, which is consistent with the low life expectancy for males in Tonga over this period. As noted in the methods, Codmod distributions could not be derived for female deaths due to the high adult mortality.

## Discussion

Certificates were not available for 54% of the reported deaths in 2008 despite the current MoH policy [9] requiring certification of all deaths. Current tabulation practices based on the immediate cause of death have contributed to a lack of reliable evidence for decision-making in Tonga. Causes of death such as ischemic heart disease and diabetes were underrepresented in these original tabulations. The tabulated cause was found to fall within the same cause aggregated to ICD list 1 as the underlying cause of death in 63% of instances

Despite data entry procedures potentially moving contributory causes into the causal sequence, the overall effect of this on selection of underlying cause is likely to be minor, other than for diabetes, if ICD selection rules are rigorously applied. The general principle of ICD rules require selection of the lowest completed line on Part I of the certificate as the underlying cause of death only if the condition "could have given rise to all the conditions entered above it" [10], thereby excluding the contributory cause in most cases even if recorded in the causal sequence of Part I of the certificate. However, while the ICD rules allow cardiovascular conditions to be considered as a consequence of diabetes, the actual recording of diabetes in Part 1 or Part 2 in such cases is driven by individual certifier preferences rather than an internationally recognized causal relationship between diabetes and cardiovascular disease. This affects comparability of underlying cause statistics between populations and across time. In this study, the process of generating two tabulations based on different assumptions of how contributory causes were treated (as described in the methods) in effect also reflects this potential ambiguity in the results [21]. The increases in cause-specific mortality from 2001-2004 to 2005-2008 from noncommunicable diseases, including cardiovascular diseases and diabetes, are significant, with the range of estimates from each period not overlapping, indicating that increases in cause-specific mortality from diabetes are not an artifact of changes in coding conventions. Noncommunicable diseases are clearly leading the high rates of adult mortality, although injury and external causes remain the leading cause of death in 15- to 24-year-olds. Current tabulations of data in Tonga code external cause by injury type rather than cause (such as fall, motor vehicle accident, etc.), and there was insufficient detail in many cases (both on the certificate or medical record) to assign a more specific cause category. Injury mortality data are thus currently of limited use to target public health interventions, and doctors should be encouraged to record more detail for these events on the medical certificate of death.

The data for 2008 indicate 93% of the final assigned causes matched those listed on the medical certificate of death (ICD list 1). There were, however, some clear patterns of misreporting on the certificate, primarily for children, where additional information from the record could have led to a more specific diagnosis. Inconsistencies were also noted in the reporting of diabetes and cardiovascular disease, with these conditions being the most likely underlying causes from the medical certificate to be assigned to other causes by the medical record review. This demonstrates the importance of certifying doctors referring to the medical record, particularly in cases due to chronic disease, or where death is attributed to an ill-defined or unknown cause. There is also a critical need to improve the quality of medical records and availability of this information to the certifying doctor. Even following review, 6% of deaths remained assigned to unknown causes. In part, this may reflect the community attendance (or lack thereof) at the hospital and subsequently limited opportunity for data collection. However, 28% of the deaths for which no medical record could be located died at the hospital and therefore should have had a medical record available. In part these missing records may be due to cases that were "dead on arrival" at the hospital and therefore certified without a record being generated. For the remainder, as the study was conducted some months after the conclusion of 2008, these missing records cannot be accounted for by delays in transfer of the record from the ward to the medical records office. This highlights a critical need to improve data management procedures to ensure that data are not lost once collected and that all records can be accessed centrally.

The medical record review of deaths that occurred in 2008 was conducted in accordance with the principles set out by Johanssen et al. [14] to ensure the transparency and reproducibility of medical record review studies. However, this work varied from previous applications of this technique as the small population and health service arrangements meant that an independent review in-country was not possible, thus introducing an additional step of extracting data from the medical record rather than having a physician review the record directly. Previous studies have also started from the presumption of a reference standard, where one source is assumed to be more accurate than the other (i.e., the certificate when reviewing verbal autopsy findings or the medical record when reviewing the medical certificate), which was not appropriate in this setting. In order to ensure the integrity of the study given these design differences, data extractions from the medical record were guided by a standardized form and instruction manual [12], extractions were completed by nurses with a good understanding of medical terminology, and forms were routinely reviewed by the project team for quality. Causes of death were coded according to ICD rules [10] and collated to the recommended tabulations in the ICD [10]. The approach used in this study allowed the flexibility to ensure the most reliable data from either the medical record or medical certificate of death were used to assign underlying cause. This was an important consideration given the unknown quality of cause of death data in both the medical certificates of death and medical records at the outset of the study. A complete review of information in the medical record, rather than the principal diagnosis as recorded on the discharge summary was used for comparison to the medical certificate, as the principal diagnosis is the reason for the admission and is frequently a manifestation of an underlying cause that may not be recorded. For example, a patient admitted for congestive heart failure and who subsequently dies would have this recorded as the principal diagnosis rather than the underlying cardiopathy that led to this condition. As cause of death data were obtained as ICD codes it was not possible to differentiate whether errors were introduced during original certification or coding, and a further coding audit may also prove useful.

There is very little information currently published on causes of death in Tonga. Of the published data available, the WHO Child Health Epidemiology Reference Group indicates similar estimates of proportional mortality for 2008 in children under 5 years of age from external causes (7%) but higher mortality from respiratory diseases (pneumonia) (17%) and congenital abnormalities (17%) compared to this study [22]. In adults, while significant uncertainty remains due to both previous data entry practices and the small number of deaths in the study, the findings demonstrate that diabetes is a much greater public health issue in Tonga than previously reported. Age-standardized mortality in Tonga from diabetes for 2005 to 2008 is estimated at 94 to 222 deaths per 100,000 for males and 98 to 190 for females. This is significantly higher than the estimated age-standardized rates of 16 (males) and 11 (females) deaths per 100,000 population respectively for New Zealand (2008) [23] and previous GBD estimates for Tonga (2008) of 40 (males) and 53 (females) deaths per 100,000 [19], although the latter may have been based on tabulations of immediate cause. The high estimates of cause-specific mortality from diabetes in Tonga are, however, broadly consistent with previous studies for New Zealand's Maori, a group that also demonstrates high mortality from diabetes at a lower prevalence of disease. A 2004 survey estimated prevalence of diabetes in the Tongan population at 18% (based on self-report) [24], while prevalence (by self-report) in the Maori population was estimated at 8% for males and 12% for females in 2002 [25]. Age-standardized mortality from diabetes for Maori males was estimated at 61 deaths per 100,000 and 39 deaths per 100,000 for females in 2004 [25]. While lower than the estimates of age-standardized mortality from diabetes in Tonga, the Maori figures are based on the older WHO standard population (the Segi age distribution) [18] and would be substantially higher if the newer WHO world standard population (which has a higher proportion of the population in the adult age groups) had been used, as applied in this study.

A study of diabetic patients in New Zealand also found that Tongan patients tended to have poorer compliance with treatment and a more fatalistic attitude to their illness [26]. This may also be a factor in the high mortality rates seen in Tonga and is consistent with the high proportion of medical record extracts collected in this study in which the case's diabetes was referred to as "uncontrolled" and "unmanaged."

Age-standardized death rates for ischemic heart disease and neoplasms were substantially higher in males in Tonga compared to Australia and New Zealand. For Tongan males, age-standardized mortality from ischemic heart disease is estimated at 139 to 210 deaths per 100,000 (2005-2008), compared with 97 in New Zealand [23] and 78 in Australia [19] in 2008. Age-standardized mortality in males from neoplasms is estimated at 189 to 255 deaths per 100,000 (2005-2008) in Tonga compared with 155 deaths per 100,000 in New Zealand and 146 in Australia in 2008 [19]. Age-standardized mortality from ischemic heart disease and neoplasms for females (2005-2008) was very similar to estimates reported for New Zealand [23] and slightly higher than those for Australia in 2008 [19]. The higher age-standardized rates for cardiovascular disease and neoplasms would contribute to the relatively low life expectancy for males in Tonga (60.4 to 64.2 years, 2005-2009) [5], highlighting the critical importance of addressing chronic diseases in Tonga to improve life expectancy. Age-standardized rates for ischemic heart disease remain below those seen in Australia (472 deaths per 100,000 for males and 246 deaths per 100,000 for females at the height of the cardiovascular disease epidemic (1968)) [27].

The high age-standardized mortality rates from cardiovascular diseases are consistent with the limited information on noncommunicable disease risk factors available for Tonga. A 2004 survey found that 67% of adults were obese (body mass index ≥ 30 kg/m^2^), with 23% of adults affected by hypertension (systolic blood pressure ≥ 140 mmHg and/or diastolic blood pressure ≥ 90 mmHg or currently on medication) [24]. The higher age-standardized rate of lung cancer in males of 34.4 to 49.0 deaths per 100,000 compared with 16.5 to 23.0 for females (2005-2009) reflects significantly higher smoking prevalence amongst males (64%) than females (14%), as recorded in a population-based survey undertaken in 1992 [28]. These rates of lung cancer are substantially higher than previous estimates from the GBD study (2008) of cause-specific mortality of 15 deaths per 100,000 for males and eight deaths per 100,000 for females [19].

Increases in age-standardized mortality from infectious diseases were largely accounted for by cause-specific mortality from hepatitis B and septicemia and indicate a need for further consideration for early universal vaccination for hepatitis B and improved reporting of underlying conditions that may result in septicemia (such as diabetes) on the medical certificate.

## Conclusion

This paper presents the first published comparative validation study of cause of death data in a Pacific Island country and demonstrates clearly the impact of noncommunicable diseases on life expectancy in Tonga. This is supported by the significant increase in age-specific mortality from a range of noncommunicable diseases noted between the two periods, including diabetes, lung cancer, and cardiovascular disease, although specific subcategories of disease such as ischemic heart disease are more likely to be affected by quality issues in the earlier period (prior to the new MoH policy on certification) and therefore will be less reliable than the broader categories. Although Tonga was the first country in the region to develop a strategy to combat noncommunicable diseases [29], more clearly needs to be done to address this growing health issue.

This research also outlines the potential for improving cause of death data available in Tonga. Changes have already been made to data entry and tabulation practices in order to separate contributory causes in Part II of the certificate from the causal sequence in Part I and to ensure tabulations from the MoH database are generated from the underlying cause of death. However, despite the potential for further improvements in cause of death data, there are significantly more data on cause of death available from Tonga than are routinely reported or known to international agencies, and it is imperative that health planners are able to access this information.

## Competing interests

ADL is an Editor-in-Chief of *Population Health Metrics*.

## Authors' contributions

KC designed the study, developed the study forms and procedures, conducted initial training of data extractors, reviewed trial extraction forms, arranged coding and collation of data, analyzed the collected data, and drafted the manuscript; SH coordinated data extraction in Tonga and provided significant input to the manuscript; CR assisted with reviewing trial extraction forms and revised the manuscript critically for important intellectual content; SA reviewed the extracted data from both countries to allocate cause of death from the medical record and provided significant input into the discussion; RR provided the second review of deaths for which cause was originally assigned as unknown and critically revised the manuscript. ADL and RT conceived and coordinated the broader study under which this project was undertaken, reviewed and contributed significantly to the interpretation of results, and revised the manuscript critically for important intellectual content. RT also made substantial contributions to the study design. All authors read and approved the final manuscript.

## Supplementary Material

Additional file 1 **World Health Organization**. *International Statistical Classification of Diseases and Related Health Problems*. Tenth Revision. Geneva: 2007 [cited 26/11/2010] http://www.who.int/classifications/icd/en/. Click here for file

Additional file 2 **Source data for selection of final cause-of-death in matched records, and reason for selection; Tongatapu (2008)**. Click here for file
