# Supplementary material for: Causes of death in Tonga: quality of certification and implications for statistics
Source: Popul Health Metr. 2012 Mar 5;10:4. doi: 10.1186/1478-7954-10-4 (PMC3378436; doi:10.1186/1478-7954-10-4)
Supplement: Additional file 1 — World Health Organization. International Statistical Classification of Diseases and Related Health Problems. Tenth Revision. Geneva: 2007 [cited 26/11/2010] http://www.who.int/classifications/icd/en/. [file 1478-7954-10-4-S1.DOC]

Appendix 1


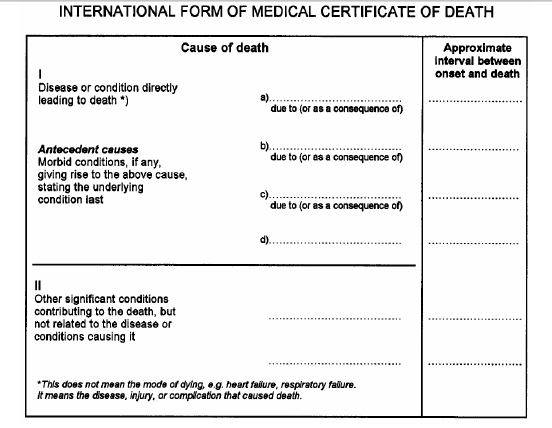


1. World Health Organization. *International Statistical Classification of Diseases and Related Health Problems*. Tenth Revision. Geneva: 2007 [cited 26/11/2010] http://www.who.int/classifications/icd/en/
