# Supplementary material for: Causes of death in Tonga: quality of certification and implications for statistics
Source: Popul Health Metr. 2012 Mar 5;10:4. doi: 10.1186/1478-7954-10-4 (PMC3378436; doi:10.1186/1478-7954-10-4)
Supplement: Additional file 2 — Source data for selection of final cause-of-death in matched records, and reason for selection; Tongatapu (2008). [file 1478-7954-10-4-S2.DOC]

**Appendix 2: Source data for selection of final cause-of-death in matched records, and reason for selection; Tongatapu (2008)**

|  | **Records** | | |  |
| --- | --- | --- | --- | --- |
| **Certificate used as final diagnosis** | **179** | **/** | **29** | **Medical Record used as final diagnosis** |
|  |  |  |  |  |
| Medical record could not assign cause-of-death | 96 | / | 16 | Death assigned as due to “unknown” cause on certificate |
|  |  |  |  |  |
| Death due to injury (not in record) | 1 | / | 4 (1 + 3) | Death due to current injury not on certificate  or More specific details of injury included in medical record |
|  |  |  |  |  |
| Diagnosis from medical record of limited certainty | 78 | / | 9 | Certificate recorded death as non-specific cause |
|  |  |  |  |  |
| Underlying cause-of-death from certificate could lead to underlying cause identified in medical record | 4 | / | 0 |  |
